# Dietary Magnesium Intake and Metabolic Syndrome in the Adult Population: Dose-Response Meta-Analysis and Meta-Regression

**DOI:** 10.3390/nu6126005

**Published:** 2014-12-22

**Authors:** Sang-Yhun Ju, Whan-Seok Choi, Sun-Myeong Ock, Chul-Min Kim, Do-Hoon Kim

**Affiliations:** 1 Department of Family Medicine, Yeouido St. Mary’s Hospital, College of Medicine, The Catholic University of Korea, 10, 63-ro, Yeongdeungpo-gu, Seoul 150-713, Korea; E-Mail: soulfree@catholic.ac.kr; 2 Department of Family Medicine, Seoul St. Mary’s Hospital, College of Medicine, The Catholic University of Korea, 22 Banpo-daero, Seocho-gu, Seoul 137-701, Korea; E-Mails: fmchs@dreamwiz.com (W.-S.C.); musofm@unitel.co.kr (C.-M.K.); 3 Department of Family Medicine, Korea University Ansan Hospital, 516, Gojan 1-Dong, Danwon-Gu, Ansan-Si Gyeonggi-Do 425-707, Korea; E-Mail: kmcfm@hanmail.net

**Keywords:** magnesium intake, metabolic syndrome, meta-analysis, meta-regression

## Abstract

Increasing evidence has suggested an association between dietary magnesium intake and metabolic syndrome. However, previous research examining dietary magnesium intake and metabolic syndrome has produced mixed results. Our objective was to determine the relationship between dietary magnesium intake and metabolic syndrome in the adult population using a dose-response meta-analysis. We searched the PubMed, Embase and the Cochrane Library databases from August, 1965, to May, 2014. Observational studies reporting risk ratios with 95% confidence intervals (CIs) for metabolic syndrome in ≥3 categories of dietary magnesium intake levels were selected. The data extraction was performed independently by two authors, and the quality of the studies was evaluated using the Risk of Bias Assessment Tool for Nonrandomized Studies (RoBANS). Based on eight cross-sectional studies and two prospective cohort studies, the pooled relative risks of metabolic syndrome per 150 mg/day increment in magnesium intake was 0.88 (95% CI, 0.84–0.93; *I*^2^ = 36.3%). The meta-regression model showed a generally linear, inverse relationship between magnesium intake (mg/day) and metabolic syndrome. This dose-response meta-analysis indicates that dietary magnesium intake is significantly and inversely associated with the risk of metabolic syndrome. However, randomized clinical trials will be necessary to address the issue of causality and to determine whether magnesium supplementation is effective for the prevention of metabolic syndrome.

## 1. Introduction

Metabolic syndrome is characterized by hyperinsulinemia with underlying insulin resistance, as well as a variety of other cardiovascular risk factors, including impaired glucose regulation, elevated levels of triglycerides, decreased levels of high-density lipoprotein cholesterol, increased blood pressure and centrally-distributed obesity [1]. Metabolic syndrome [2] has received increased attention in the past year. Given that metabolic syndrome is strongly linked to cardiovascular events [3], cancer and mortality [4], preventing metabolic syndrome is important to public health.

Dietary and lifestyle modifications are widely perceived to play an important role in reducing metabolic syndrome [5]. Magnesium is an essential trace mineral for the human body that plays a key role in all energy-dependent transport systems, glycolysis and oxidative energy metabolism [6,7]. Foods rich in magnesium include whole grains, spinach, nuts, legumes and potatoes [8]. Magnesium deficiency, either from inadequate intake, excess excretion or altered homeostasis, is often suspected to be associated with the initiation of many symptoms and diseases [9]. Lifestyle factors (e.g., poor nutrition and excess alcohol intake), certain types of medications (e.g., diuretics) and lower mineral content [10,11] in commonly-eaten foods (e.g., fruits and vegetables) have led to an increase in studies evaluating the potential link between magnesium deficiency and numerous health conditions [12].

Low serum magnesium levels have been associated with risk factors of metabolic syndrome, such as hyperglycemia, hypertension, hypertriglyceridemia and insulin resistance [13]. Additionally, waist circumference is independently associated with hypomagnesemia [14]. Furthermore, several studies [15,16,17] suggested that serum magnesium levels are independently associated with metabolic syndrome. Hence, a high dietary magnesium intake may potentially reduce the risk of metabolic syndrome. However, recent epidemiological studies have been conducted to investigate the association between magnesium intake and metabolic syndrome, with inconsistent results. Several epidemiological studies [18,19,20] indicated that deficient magnesium intake may be an independent risk factor for metabolic syndrome; however, other studies [21,22] have reported no significant association.

Therefore, we conducted a meta-analysis of observational studies for the following purposes: (1) to assess the association between dietary magnesium intake and metabolic syndrome; (2) to evaluate a dose-response pattern of dietary magnesium intake on the risk of metabolic syndrome; and (3) to examine the association according to the characteristics of study designs and populations.

## 2. Materials and Methods

Following generally-accepted methodology recommendations, the meta-analysis was performed according to the Preferred Reporting Items for Systematic Review and Meta-Analyses statement (Table S1: PRISMA checklist) [23]. This study was registered at both the ClinicalTrials.gov Protocol Registration System (NCT02151227), https://clinicaltrials.gov/ct2/show/NCT02151227?term=NCT02151227&rank=1 and the International Prospective Register of Systematic Review, www.crd.york.ac.uk/PROSPERO/ (CRD42014010545).

### 2.1. Literature Search

A medical librarian with a Master’s degree and experience in systematic reviews participated in the search strategy design process. We searched PubMed, the Cochrane Library and Embase databases via Elsevier from August, 1965, to May, 2014. A PubMed search for studies on magnesium and metabolic syndrome was conducted without restrictions, by combining synonymous or related search terms for magnesium and metabolic syndrome. The keywords used in the PubMed search were converted to search tags for the Cochrane Library and EMBASE (Table S2: search strategies). Furthermore, manual searches of the bibliographies of relevant articles were performed to identify additional studies.

### 2.2. Excluded Studies

In total, 452 articles were found in our initial search. Using an automated reference manager facility, 128 duplicates were removed, leaving a database containing 324 references. In the first round of screening on the basis of the title or abstract, we excluded 266 articles for at least one of the following reasons: did not study magnesium exposure or use metabolic syndrome as an outcome; was a duplicate study; was a nonhuman study; was a non-original study, such as a review, editorial, letters or commentary. In the second round of screening on the basis of full-text articles, we excluded 49 articles because these did not meet the inclusion criteria: no full text available (4 articles), no relevant studies (25 articles), no original articles (13 articles), data not available (3 articles) and duplicates (4 articles) (Table S3: excluded studies). Lastly, 10 independent observational studies (extracted from 9 articles), including 8 cross-sectional studies and 2 prospective cohort studies, were included in the meta-analysis (Figure S1: flow diagram for the search strategy and study selection process). Furthermore, manual searches of the bibliographies of relevant articles were performed to identify additional studies.

### 2.3. Validity Assessment

The quality of the primary studies was evaluated using the Risk of Bias Assessment Tool for Nonrandomized Studies (RoBANS), which showed moderate reliability, promising feasibility and validity [24]. RoBANS consists of the following 6 domains: the selection of the participants, confounding variables, measurement of exposure, blinding of outcome assessments, incomplete outcome data and selective outcome reporting.

### 2.4. Data Extraction

Two investigators (Sang-Yhun Ju and Do-Hoon Kim, co-authors of the present study, independently extracted the data from the original reports. The adjusted risk estimates that reflected the most comprehensive control were extracted to avoid potential confounding variables. The following information was extracted: study characteristics (study name, study design and location, first author’s surname, publication year and adjusted covariates), participants’ characteristics (age, sex, sample size and sample source), methods of dietary magnesium assessment and the definition of metabolic syndrome used.

### 2.5. Statistical Analysis

Summary risk ratios were calculated by pooling the study-specific estimates using a random-effects model, which considered both within-study and between-study variations [25]. We also performed a random-effects meta-regression model to assess the overall linear relationship between dietary magnesium intake and metabolic syndrome with weights, based on the inverse of variances [26].

For the dose-response meta-analysis, we used generalized least-squares regression (Stata GLST command), which considered the correlation between estimates for different exposure levels [27], to compute study-specific slopes (linear trends). This method required that the number of cases and controls and the risk ratio with its variance estimate for at least three quantitative exposure categories were known. For the studies that did not provide this information, we estimated the dose-response slopes using variance-weighted least-squares regression (Stata VWLS command). Both methods (GLST and VWLS) required median or mean exposure for each category of magnesium intake level. The mid-point of each category of magnesium intake was calculated, and half the width of the adjacent category was used to define the corresponding point for open-ended categories.

The reported HRs and ORs were assumed to approximate the same measure of RRs [28]. Where studies reported RRs with differing degrees of adjustment for other risk factors, the maximum adjusted estimate was used. Dose responses of dietary magnesium were standardized across studies to 150 mg/day, based on the interquartile range between the bottom and top quartiles across all studies of 148 mg/day for dietary magnesium. The RRs for different increments can be calculated from our data. Although the magnesium intake in our included studies was expressed in different units, we rescaled it to milligrams (mg) per day. When the exposures were expressed in mg/1000 kcal, we converted it to mg/day using the average energy intake (kcal/day) reported in that article [19]. For the study reporting intakes in mg/kg/day [29], the intakes in mg/day were estimated using the average body weight of Taiwan elderly individuals reported in that article [30].

The subgroup meta-analyses were conducted according to the pre-specified study-level characteristics using a fixed-effects meta-analysis. The sources included location, study design (cohort, cross-sectional), sex, age, sample size, sample source (population-based, hospital-based), the dietary assessment method, the definition of metabolic syndrome used and the risk of bias.

The statistical heterogeneity among the studies was assessed using *I*^2^ statistics [31]. We defined low, moderate and high heterogeneity as *I*^2^ values of 25%, 50% and 75%, respectively. Heterogeneity was assessed by comparing the results from studies grouped according to the pre-specified study-level characteristics using meta-regression. We also conducted sensitivity analyses to evaluate the potential sources of heterogeneity in the analyses. The publication bias was evaluated using Egger’s test and Begg’s test. In the presence of publication bias, the *p*-values for Egger’s test and Begg’s test are less than 0.05. All of the statistical analyses were performed using Stata software, version 13.0 (Stata Corp., College Station, TX, USA).

## 3. Results

### 3.1. Study Characteristics, Quality and Bias Assessment

We identified 10 articles, including nine [18,19,20,21,22,29,32,33,34] that investigated the association between dietary magnesium intake and metabolic syndrome and one article [33] that reported results separately for gender (men and women).

The studies included in this meta-analysis are summarized in Table 1. A total of 30,092 participants were reported in 10 observational studies, including eight cross-sectional studies [18,20,21,29,32,33,34] and two prospective cohort studies [19,22]. Eight population-based studies [18,19,20,21,32,33,34] involving healthy populations were included, and two hospital-based studies recruited patients with elderly type 2 diabetes [29] and recipients of living-donor kidney transplant [22]. Four studies were conducted in the United States [18,19,20,34], four in Asia [22,29,32,33] and one in Europe [21]; all of the studies were published in the 2000s. The participants’ mean age ranged from 18 years to 72 years. Four studies [18,19,21,22] defined metabolic syndrome using the Third Report of the National Cholesterol Education Program (NCEP) Expert Panel on Detection, Evaluation and Treatment of High Blood Cholesterol in Adults (ATP-III), and six studies [20,29,32,33,34] assessed metabolic syndrome using the modified NCEP ATP-III and International Diabetes Foundation. The assay method of dietary magnesium intake varied across the studies. Four studies [19,20,21,22] used the Food Frequency Questionnaire (FFQ); five studies [18,29,32,33] used 24-h dietary recalls; and one study [34] used a consecutive three-day food record.

**Figure 1:**
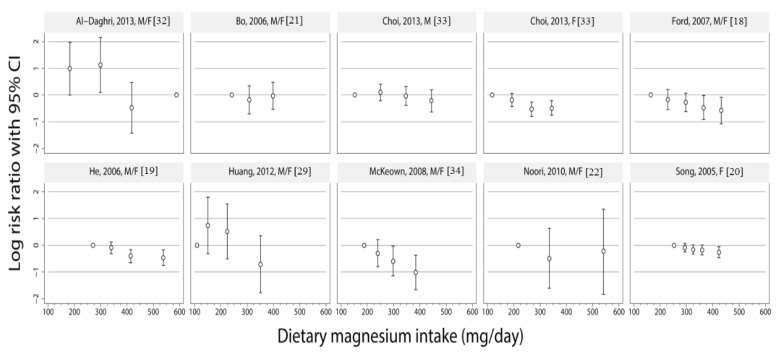
Study-specific risks ratios (RRs) and 95% CIs of metabolic syndrome risk according to study-specific levels of dietary magnesium intake. Depending on available information, the median, midpoints or means of the categories were used for defining study-specific levels of magnesium intake categories (mg/day). The vertical axis is on a log scale.

The reported risk estimates for the association of dietary magnesium intake levels with metabolic syndrome are illustrated in Figure 1. There appeared to be an approximately inverse linear relationship of magnesium intake levels and metabolic syndrome in all of the studies, except for the studies of Al-Daghri *et al.* [32], Bo *et al.* [21] and Noori *et al.* [22], which found no association between magnesium intake and metabolic syndrome. For all other studies, the group with the highest magnesium intake levels had the lowest metabolic syndrome risk.

**Table 1:** Characteristics of studies included in the meta-analysis on the association of dietary magnesium intake with metabolic syndrome.

Author, Year	Country (Study)	Population	Baseline Population	Participant Cases	Sex	Assessment (Exposure)	Key Set of Covariates ^1^
		Study Design			Mean Age	Definition (Outcome)	
Song *et al.*, 2005 [20]	USA (WHS)	Population-based	Healthy	9907	F	FFQ	+
		Cross-sectional		2418	52	Modified NCEP-ATP III	
Bo *et al.*, 2006 [21]	Italy	Population-based	Healthy	1653	M and F	FFQ	+
		Cross-sectional		384	54	NCEP-ATP III	
He *et al.*, 2006 [19]	USA (CARDIA)	Population-based	Healthy	4637	M and F	FFQ	++
		Prospective cohort		608	25	NCEP-ATP III	
Ford *et al.*, 2007 [18]	USA (NHANES III)	Population-based	Healthy	7669	M and F	24-h recall	+
		Cross-sectional		4965	44	NCEP-ATP III	
McKeown *et al.*, 2008 [34]	USA	Population-based	Healthy	535	M and F	3-D food record	++
		Cross-sectional		212	72	Modified NCEP-ATP III	
Noori, 2010 [22]	Iran	Hospital-based	Renal-transplant recipient	160	M and F	FFQ	+
		Prospective cohort		58	40	NCEP-ATP III	
Huang *et al.*, 2012 [29]	Taiwan	Hospital-based	Diabetes	210	M and F	24-h recall	++
		Cross-sectional		156	72	NCEP-ATP III	
Al-Daghri *et al.*, 2013 [32]	Saudi Arabia	Population-based	Healthy	185	M and F	24-h recall	+
		Cross-sectional		72	26	IDF	
Choi *et al.*, 2013 [33]	Korea (KNHANES IV)	Population-based	Healthy	2084	M	24-h recall	+
				540	44	Modified NCEP-ATP III	
		Cross-sectional		3052	F		
				748	49		

Abbreviations: CARDIA, Coronary Artery Risk Development in Young Adults; CDS, Chinese Diabetes Society; FFQ, food-frequency questionnaire; IDF, International Diabetes Foundation; KNHANES, Korea National Health and Nutrition Examination Survey; NCEP-ATP III, National Cholesterol Education Program and Adult Treatment Panel III; NHANES III, The Third National Health and Nutrition Examination Survey; TLGS, Tehran Lipid and Glucose Study; WHS, Women’s Health Study; ^1^ Key set of covariates indicates age, sex, obesity, smoking, alcohol intake, exercise and calories intake: ++, the key sets of covariates were adequately confirmed and adjusted for during the analysis phase; +, although the existence of major confounding variables were confirmed, the key sets of covariates were not adequately considered during the design and analytic phases.

The risk of bias analysis revealed several areas of concern (Figure S2: assessment of the risk of bias), particularly with regard to confounding variables in two studies [32,33] that did not adjust for the key covariates, including age, sex, obesity, smoking, alcohol intake, exercise and calorie intake.

### 3.2. Meta-Regression and Dose-Response Meta-Analysis

**Figure 2:**
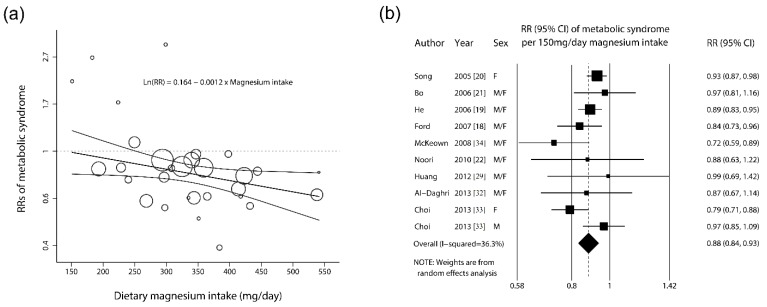
(**a**) Association between the risk of metabolic syndrome and dietary magnesium intake: dose-response meta-regression. The levels of magnesium intake (mg/day) were modeled using a linear trend with random-effects meta-regression models. The solid black line represents the weighted regression line based on variance-weighted least squares. The gray line shows the 95% CI around the regression line. The circles indicate RRs in each study. The circle size is proportional to the precision of the RR. The vertical axis is on a log scale. (**b**) Forest plots of the risks ratios (RRs) of metabolic syndrome per 150 mg/day increment in dietary magnesium intake (*n* = 30,092) using a random-effects analysis. The squares represent study-specific RR (the square sizes are proportional to the weight of each study in the overall estimate); the horizontal lines represent 95% confidence intervals (CIs); and the diamond represents the overall RR estimate with the 95% CI.

As shown in Figure 2a, for the ten epidemiological studies that assessed dietary magnesium intake, the pooled estimate indicated that magnesium intake (mg/day) was significantly and inversely associated with metabolic syndrome based on the meta-regression model, as follows:

(1) In (RR of metabolic syndrome)=0.1694−0.0012×Dose of magnesium intake(mg/day)

(2) $$Adjusted\;R^{2}=22.4\%;\;I^{2}=39.8\%;\;\tau^{2}=0.011;\;p=0.04$$

The estimated RRs of metabolic syndrome for an increase in magnesium intake of 150 mg/day for each of the 10 observational studies are shown in Figure 2b. A significant inverse association between magnesium intake and metabolic syndrome was observed in five studies [18,19,20,33,34], and a nonsignificant inverse association was found in five studies [21,22,29,32,33]. In the analysis of all of the studies, the overall RR demonstrated a statistically significant inverse association between dietary magnesium intake of an increase in 150 mg/day and metabolic syndrome (RR, 0.88; 95% CI, 0.84–0.93). There was moderate heterogeneity across studies (*I*^2^ = 36.3%). In a sensitivity analysis in which one study at a time was removed and the remaining studies were analyzed, the RR ranged from 0.87 (95% CI, 0.82–0.92) when the study by Song *et al.* [20] was excluded to 0.90 (95% CI, 0.87–0.94) when the study (on women) by Choi *et al.* [33] was excluded. No publication bias was detected with Egger’s test (*p* for bias = 0.352) or Begg’s test (*p* for bias = 0.655) (Figure S3: Begg’s funnel plots in the meta-analysis of observational studies).

### 3.3. Subgroup Analyses and Publication Bias

**Figure 3:**
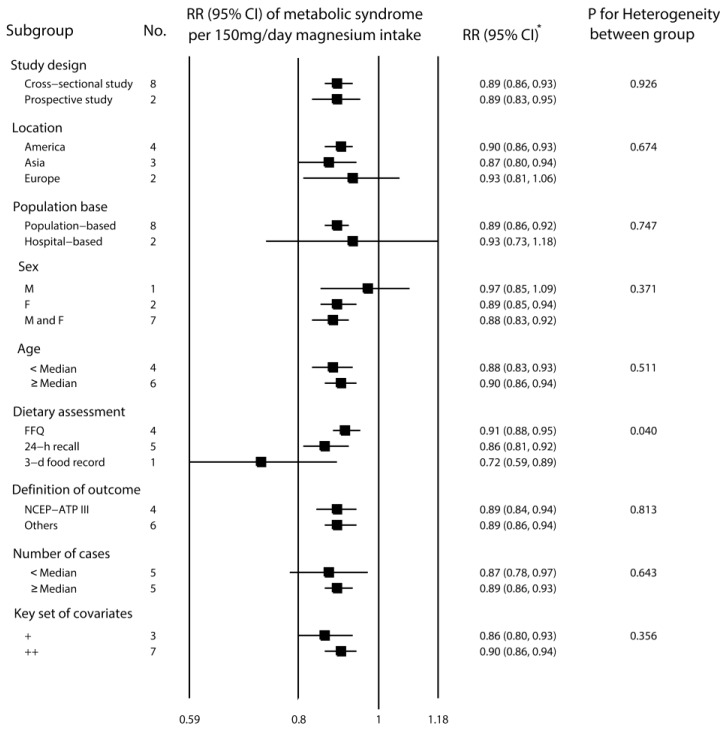
Relative risk for metabolic syndrome per 150 mg/day increment in dietary magnesium intake according to different study-level characteristics. * *p* > 0.05 from meta-regression analyses on each of the covariates; FFQ, food frequency questionnaires; NCEP-ATP III, National Cholesterol Education Program and Adult Treatment Panel III; the key sets of covariates indicate age, sex, obesity, smoking, alcohol intake, exercise and calorie intake: ++, the key sets of covariates were adequately confirmed and adjusted for during the analysis phase; +, although the existence of major confounding variables were confirmed, the key sets of covariates were not adequately considered during the design and analytic phases.

The subgroup analyses using fixed effects are shown in Figure 3. There were significant differences in dietary magnesium assessment (*p* = 0.04 for heterogeneity between groups). The corresponding RRs for studies based on FFQ, 24-h recall and 3-D food records were 0.91 (95% CI, 0.88–0.95), 0.86 (95% CI, 0.81–0.92) and 0.72 (95% CI, 0.59–0.89), respectively. In contrast, no significant group difference was found for study design, location, population base, sex, age, definition of outcome, number of cases and key sets of covariates (*p* > 0.1 for heterogeneity between groups). The univariate meta-regression analyses indicated no influence of the difference in dietary assessment, study design, location, population base, sex, age, definition of outcome, number of case and key sets of covariates on the inverse association between dietary magnesium intake and the risk of metabolic syndrome (*p* > 0.05 for all).

## 4. Discussion

This meta-analysis of observational studies showed a statistically significant inverse association between dietary magnesium intake and the risk of metabolic syndrome based on cross-sectional studies and prospective cohort studies published between 2005 and 2013. The overall estimate indicated a 12% reduction in the risk of metabolic syndrome for an increment in magnesium intake of 150 mg/day, which is approximately equivalent to the magnesium content in 2 oz. of dry roasted almonds, one cup of cooked spinach, 1.5 cup of beans, two cups of brown, long-grained cooked rice, three medium baked potatoes with skin, five medium bananas or six tablespoons of peanut butter per day [35]. Moreover, using the data on magnesium intake levels, a progressively decreasing relationship was observed for low levels of magnesium intake in the pooled analysis. There was some heterogeneity among the studies, although there were no publication bias and no differences between the subgroups, including dietary assessment, study design, location, cohort-based, sex, age, definition of outcome, number of case and key sets of covariates using meta-regression.

This dose-response meta-analysis allowed us to evaluate the risk across the entire spectrum of observed magnesium intake levels. We found a monotonically decreasing association between magnesium intake levels and the risk of metabolic syndrome in the adult population, which suggested that any incremental increase in the magnesium intake level was associated with a decreased risk in the metabolic syndrome. However, because the range of magnesium intake levels was centered at approximately 200 mg/day to 450 mg/day, the apparent dose relationship at higher levels of magnesium intake might have been weak. Therefore, this result should be interpreted cautiously.

The inverse association found in our study is supported by evidence from randomized controlled studies [36,37] that cite the role of magnesium as an important component in improving insulin resistance, a central feature of metabolic syndrome. In addition, one case-control study [15] reported an inverse association between low serum magnesium levels and metabolic syndrome. A dietary intervention study [38] found that magnesium intake was inadequate among non-diabetic individuals with metabolic syndrome and that a higher level of magnesium intake from food had a beneficial effect on insulin resistance.

The potential protective role of magnesium intake against the risk of metabolic syndrome may be related to its beneficial effects on individual components of metabolic syndrome. The experimental data suggest that magnesium is a necessary cofactor for several enzymes that play an important role in glucose metabolism, especially those involved in autophosphorylation of the β-subunit of the insulin receptor and the activity of muscle insulin tyrosine kinase [39]. A recent meta-analysis suggested that higher dietary magnesium intake was inversely associated with fasting glucose and fasting insulin in individuals free of diabetes, generally, irrespective of genetic variation at the glycemia and magnesium-related loci investigated [40].

The findings from a current meta-analysis of 22 randomized clinical trials (mean duration, 11.3 weeks) have shown that magnesium supplementation (mean dose, 410 mg) significantly decreased blood pressure by 3–4 mmHg for systolic pressure and 2–3 mmHg for diastolic pressure, which is more evident at the higher dose (≥370 mg/day) [41]. An animal study of streptozocin-induced diabetic rats found that administering magnesium sulfate for eight weeks can improve the lipid profile, decrease mesenteric fat and also decrease systolic and diastolic blood pressure, suggesting that magnesium sulfate administration decreases cardiovascular risk factors [42]. The beneficial effect of magnesium supplementation on lipid parameters has also been attributed to improving insulin sensitivity, because it normalized total cholesterol and triglyceride levels, which were impaired by a high fructose diet in rats [43].

Evidence that magnesium is directly involved in body weight regulation is lacking. The association between magnesium intake and obesity is a matter of controversy [44]. Two population-based studies have reported either a negative or no relationship between magnesium and body mass index [19,20], whereas a cross-sectional study [45] found a significant inverse association between magnesium intake and obesity or central obesity, which suggests that the inverse association may be attributable to dietary magnesium’s ability to form bonds with fatty acids in the intestine and, thus, reduce the digestible energy content of the diet [46]. Additionally, a recent study demonstrated that higher dietary magnesium intake is associated with improved insulin sensitivity and that this effect is particularly beneficial for overweight and obese individuals in the general population [47].

Within the subgroup analysis, we found that the association was attenuated in studies that assessed dietary magnesium intake with an FFQ only at baseline, which generally leads to bias in observed relative risks and loss of power to detect diet-disease relationships [48,49]. Additionally, some degree of misclassification of exposure may have weakened the strength of the association [50,51]. As an instrument for quantifying nutrient intakes, FFQ is relatively rough, although the majority of FFQs have been validated before application [52]. Such errors may have been present in other studies [53,54] that assessed dietary magnesium at baseline only, which could lead to an underestimation of the relative risk estimates. Therefore, the observed reduction in metabolic syndrome is likely a conservative estimate. 

Although our meta-analysis included only multivariable adjusted risk ratios, there was evidence of moderate heterogeneity across the observational studies. This heterogeneity could be attributable to differences in study designs, sample sizes, analytic strategies, the participants’ characteristics, the diagnostic criteria for metabolic syndrome, study quality and the measurement of magnesium intake. To account for this heterogeneity, we used random-effects models of the meta-analyses and performed subgroup analyses using fixed-effects models; however, the results were not significantly altered. Additionally, the meta-regression did not identify any statistically significant sources of heterogeneity, although statistical power for identifying heterogeneity was limited given the number of studies.

This meta-analysis had several strengths and limitations. The primary strength was that it was the first dose-response meta-analysis that examined the relationship between the various magnesium intake levels and metabolic syndrome based on a comprehensive literature survey. Furthermore, our dose-response meta-analysis revealed a linear relationship between the magnesium intake and the risk of metabolic syndrome, despite the heterogeneous categorization of magnesium intake levels in individual studies. We found no publication bias in any of the analyses. A limitation of this approach is that it depended on the means, median or midpoints of the magnesium intake categories. The estimates of risk in this approach were therefore slightly less accurate than in the individual patient data meta-analyses. Second, despite the calculation of risk estimates that reflected the greatest degrees of controlling for potential confounders, several limitations must be considered when interpreting the meta-analysis results. Because it is impossible to adjust for general health status completely, residual confounders must always be considered when interpreting the results from observational studies. Third, one limitation was the evidence of heterogeneity across the studies, particularly resulting from heterogeneous magnesium intake measurement methods. Thus, the results of this analysis should be interpreted cautiously. We lastly were not able to assess the impact of magnesium from the supplement use on metabolic syndrome because such data are limited. Furthermore, because nearly all of the dietary magnesium in the identified studies was from foods, our findings support recommendations for increasing the consumption of magnesium-rich foods rather than taking supplements.

## 5. Conclusions

In summary, lower levels of magnesium intake were associated with the risk of metabolic syndrome in observational studies, which is consistent with the results from an analysis using linear regression methods. However, the data available for levels greater than 450 mg/day of magnesium intake were sparse, and additional studies, particularly longitudinal studies, are needed to provide more in-depth analyses, more precise and stable estimates of association and a better understanding of the potential role of magnesium intake in the risk of metabolic syndrome. Randomized clinical trials will also be necessary to address the issue of causality and to determine whether magnesium supplementation is effective for preventing metabolic syndrome.

## Supplementary Files

Supplementary File 1
