# Supplementary material for: Dietary Magnesium Intake and Metabolic Syndrome in the Adult Population: Dose-Response Meta-Analysis and Meta-Regression
Source: Nutrients. 2014 Dec 22;6(12):6005–19. doi: 10.3390/nu6126005 (PMC4277012; doi:10.3390/nu6126005)
Supplement: Supplementary File 1 [file nutrients-06-06005-s001.docx]

Supplementary Information

**Table S1.** Preferred Reporting Items for Systematic Review and Meta-Analyses (PRISMA) checklist.

| **Section/Topic** | |  | | **Checklist Item** [1] | **Reported on Page** |
| --- | --- | --- | --- | --- | --- |
| **LE** | | | | |  |
| Title | | 1 | | Identify the report as a systematic review, meta-analysis or both. | 1 |
| **Abstract** | | | | |  |
| Structured Summary | | 2 | | Provide a structured summary, including, as applicable: background, objectives, data sources, study eligibility criteria, participants and interventions, study appraisal and synthesis methods, results, limitations, conclusions and implications of key findings; systematic review registration number. | 3 |
| **INTRODUCTION** | | | | |  |
| Rationale | | 3 | | Describe the rationale for the review in the context of what is already known. | 4,5 |
| Objectives | | 4 | | Provide an explicit statement of questions being addressed with reference to participants, interventions, comparisons, outcomes and study design (PICOS). | 5 |
| **METHODS** | | | | |  |
| Protocol and Registration | | 5 | | Indicate if a review protocol exists, if and where it can be accessed (e.g., web address), and if available, provide registration information, including the registration number. | 5 |
| Eligibility Criteria | | 6 | | Specify study characteristics (e.g., PICOS, length of follow-up) and report characteristics (e.g., years considered, language, publication status) used as criteria for eligibility, giving the rationale. | 6 |
| Information Sources | | 7 | | Describe all information sources (e.g., databases with dates of coverage, contact with study authors to identify additional studies) in the search and date last searched. | 5,6 |
| Search | | 8 | | Present full electronic search strategy for at least one database, including any limits used, such that it could be repeated. | 5 |
| Study Selection | | 9 | | State the process for selecting studies (*i.e.*, screening, eligibility, included in systematic review, and if applicable, included in the  meta-analysis). | 6 |
| Data Collection Process | | 10 | | Describe the method of data extraction from reports (e.g., piloted forms, independently, in duplicate) and any processes for obtaining and confirming data from investigators. | 7 |
| Data Items | | 11 | | List and define all variables for which data were sought (e.g., PICOS, funding sources) and any assumptions and simplifications made. | 7 |
| Risk of Bias in Individual Studies | 12 | | Describe the methods used for assessing the risk of bias of individual studies (including specification of whether this was done at the study or outcome level) and how this information is to be used in any data synthesis. | | 7 |
| Summary Measures | 13 | | State the principal summary measures (e.g., risk ratio, difference in means). | | 8 |

**Table S1.** *Cont.*

| Synthesis of Results | 14 | Describe the methods of handling data and combining the results of studies, if done, including measures of consistency (e.g., *I*^2^) for each meta-analysis. | 9 |
| --- | --- | --- | --- |
| Risk of Bias Across Studies | 15 | Specify any assessment of the risk of bias that may affect the cumulative evidence (e.g., publication bias, selective reporting within studies). | 9 |
| Additional analyses | 16 | Describe methods of additional analyses (e.g., sensitivity or subgroup analyses, meta-regression), if done, indicating which were pre-specified. | 9 |
| **RESULTS** | | |  |
| Study Selection | 17 | Give numbers of studies screened, assessed for eligibility and included in the review, with reasons for exclusions at each stage, ideally with a flow diagram. | 9 |
| Study Characteristics | 18 | For each study, present characteristics for which data were extracted (e.g., study size, PICOS, follow-up period) and provide the citations. | 9,10 |
| Risk of Bias within Studies | 19 | Present data on the risk of bias of each study and, if available, any outcome level assessment (see Item 12). | 10 |
| Results of Individual Studies | 20 | For all outcomes considered (benefits or harms), present, for each study: (1) simple summary data for each intervention group; (2) effect estimates and confidence intervals, ideally with a forest plot. | 9 |
| Synthesis of Results | 21 | Present results of each meta-analysis done, including confidence intervals and measures of consistency. | 10,11 |
| Risk of Bias Across Studies | 22 | Present results of any assessment of the risk of bias across studies (see Item 15). | 10 |
| Additional Analysis | 23 | Give results of additional analyses, if done (e.g., sensitivity or subgroup analyses, meta-regression (see Item 16)). | 10,11 |
| **DISCUSSION** | | |  |
| Summary of Evidence | 24 | Summarize the main findings, including the strength of evidence for each main outcome; consider their relevance to key groups (e.g., healthcare providers, users and policy makers). | 12 |
| Limitations | 25 | Discuss limitations at the study and outcome level (e.g., the risk of bias) and at the review level (e.g., incomplete retrieval of identified research, reporting bias). | 14,15 |
| Conclusions | 26 | Provide a general interpretation of the results in the context of other evidence and implications for future research. | 15 |
| **FUNDING** | | |  |
| Funding | 27 | Describe sources of funding for the systematic review and other support (e.g., supply of data) and the role of funders in the  systematic review. | 16 |

**Table S2.** Database literature search.

| **Search** | **Query** | **Items Found** |
| --- | --- | --- |
| No. | PubMed | Results |
| 1 | “Magnesium”(MeSH) | 60,943 |
| 2 | “Magnesium Compounds”(MeSH) | 13,667 |
| 3 | Magnesium | 90,908 |
| 4 | MgCl_2_ | 12,917 |
| 5 | Brucite | 1831 |
| 6 | Magnesia | 2679 |
| 7 | Amosite | 612 |
| 8 | Cummingtonite-Grunerite | 605 |
| 9 | “Talcum Powder” | 127 |
| 10 | “Talcum Powders” | 5 |
| 11 | Talc | 2666 |
| 12 | “Mg(OH)_2_” | 222 |
| 13 | (1 or 2 or 3 or 4 or 5 or 6 or 7 or 8 or 9 or 10 or 11 or 12) | 98,094 |
| 14 | “Metabolic Syndrome X”(Mesh) | 18,568 |
| 15 | “Metabolic Syndrome” | 31,811 |
| 16 | “Metabolic Syndromes” | 338 |
| 17 | “Insulin Resistance Syndrome X” | 10 |
| 18 | “Dysmetabolic Syndrome X” | 2 |
| 19 | “Reaven Syndrome X” | 18,706 |
| 20 | “Metabolic Cardiovascular Syndrome” | 53 |
| 21 | “Metabolic Cardiovascular Syndromes” | 15,343 |
| 22 | (14 or 15 or 16 or 17 or 18 or 19 or 20 or 21) | 34,420 |
| 23 | (13 and 22) | 154 |
| 24 | (13 and 22) and Systematic Reviews | 4 |
| No. | Embase | Results |
| 1 | ‘Magnesium’/Exp or Magnesium | 129,978 |
| 2 | ‘Magnesium Derivative’/Exp | 2049 |
| 3 | ‘Magnesium Chloride’/Exp | 4756 |
| 4 | ‘Magnesium Hydroxide’/Exp | 2432 |
| 5 | ‘Magnesium Oxide’/Exp | 2853 |
| 6 | ‘Magnesium Silicate’/Exp | 398 |
| 7 | ‘Magnesium Sulfate’/Exp | 12,293 |
| 8 | MgCl_2_ | 1102 |
| 9 | ‘Brucite’/Exp or Brucite | 2471 |
| 10 | ‘Magnesia’/Exp or Magnesia | 3172 |
| 11 | ‘Amosite’/Exp or Amosite | 844 |
| 12 | ‘Cummingtonite-Grunerite’ | 8 |
| 13 | ‘Talcum Powder’/Exp or ‘Talcum Powder’ | 3723 |
| 14 | ‘Talc’/Exp or Talc | 4518 |
| 15 | ‘Mg(OH)_2_’ | 290 |
| 16 | ‘Magnogene’/Exp or Magnogene | 4756 |
| 17 | (1 or 2 or 3 or 4 or 5 or 6 or 7 or 8 or 9 or 10 or 11 or 12 or 13 or 14 or 15 or 16) | 135,445 |
| 18 | ‘Metabolic Syndrome X’/Exp | 46,476 |
| 19 | ‘Metabolic Syndrome X’/Exp or ‘Metabolic Syndrome X’ | 46,639 |
| 20 | ‘Metabolic X Syndrome’ | 37 |
| 21 | ‘Insulin Resistance Syndrome X’ | 13 |
| 22 | ‘Dysmetabolic Syndrome X’ | 3 |
| 23 | ‘Metabolic Cardiovascular Syndrome’ | 65 |
| 24 | ‘Metabolic Syndrome’/Exp or ‘Metabolic Syndrome’ | 56,479 |
| 25 | (18 or 19 or 20 or 21 or 22 or 23 or 24) | 56,552 |
| 26 | 17 and 25 | 289 |
| 27 | 17 and 25 and ‘Systematic Review’/De | 5 |

**Table S2.** *Cont.*

| No. | Cochrane Library | Results |
| --- | --- | --- |
| 1 | MeSh Descriptor: (Magnesium) Explode All Trees | 1045 |
| 2 | MeSH Descriptor: (Magnesium Compounds) Explode All Trees | 1019 |
| 3 | Magnesium: ti, ab, kw (Word Variations Have Been Searched) | 3737 |
| 4 | MgCl_2_: ti, ab, kw | 16 |
| 5 | Brucite: ti, ab, kw | 0 |
| 6 | Magnesia: ti, ab, kw | 13 |
| 7 | Amosite: ti, ab, kw | 3 |
| 8 | Cummingtonite-Grunerite: ti, ab, kw | 0 |
| 9 | Talcum Powder: ti, ab, kw | 6 |
| 10 | Talcum Powders: ti, ab, kw | 0 |
| 11 | Talc: ti, ab, kw | 103 |
| 12 | Mg(OH)_2_: ti, ab, kw | 15 |
| 13 | 1 or 2 or 3 or 4 or 5 or 6 or 7 or 8 or 9 or 10 or 11 or 12 | 3851 |
| 14 | MeSH Descriptor: (Metabolic Syndrome X) Explode All Trees | 906 |
| 15 | Metabolic Syndrome: ti, ab, kw | 1810 |
| 16 | Metabolic Syndromes: ti, ab, kw | 3 |
| 17 | Insulin Resistance Syndrome X: ti, ab, kw | 0 |
| 18 | Dysmetabolic Syndrome X: ti, ab, kw | 0 |
| 19 | Reaven Syndrome X: ti, ab, kw | 0 |
| 20 | Metabolic Cardiovascular Syndrome: ti, ab, kw | 2 |
| 21 | Metabolic Cardiovascular Syndromes: ti, ab, kw | 0 |
| 22 | Metabolic Syndrome X: ti, ab, kw | 1194 |
| 23 | 14 or 15 or 16 or 17 or 18 or 19 or 20 or 21 or 22 | 1812 |
| 24 | 13 and 23 (Systematic Reviews) | 9 |

No., number.

**Table S3.** Reference list of papers excluded in the meta-analysis.

| (1) No full text available [2–5] |
| --- |
| (2) No relevant studies [6–30] |
| (3) No original article [31–43] |
| (4) Duplicates [44–47] |
| (5) Data no available [48–50] |

**Figure S1.** Flow diagram for search strategy and study selection process [1].

Records identified through database searching
(*n* = 452)

## Screening

## Included

## Eligibility

## Identification

Additional records identified through other sources
(*n* = 0)

Records after duplicates removed
(*n* = 324)

Records screened
(*n* = 58)

Records excluded
(*n* = 266)

Full-text articles assessed for eligibility
(*n* = 9)

Full-text articles excluded, with reasons
(*n* = 49)

Studies included in qualitative synthesis
(*n* = 9)

Studies included in quantitative synthesis (meta-analysis)
(*n* = 10) ^1^

^1^ Ten independent observational studies (extracted from nine articles [45–47,51–56]), including eight cross-sectional studies and two cohorts studies were included in the meta-analysis.

**Figure S2.** Assessment of the risk of bias.

| **Year** | **2005** | **2006** | **2006** | **2007** | **2008** | **2010** | **2012** | **2013** | **2013** |
| --- | --- | --- | --- | --- | --- | --- | --- | --- | --- |
| **First author** | **Song [51]** | **Bo [52]** | **He [45]** | **Ford [53]** | **McKeown [54]** | **Noori [55]** | **Huang [46]** | **Al-Daghri [47]** | **Choi [56]** |
| Selection of participants | + | + | + | + | + | + | + | + | + |
| Confounding variables | + | + | + | + | + | + | + | − | − |
| Measurement of exposure | + | + | + | + | + | + | + | + | + |
| Blinding of outcome assessments | + | + | + | + | + | + | + | + | + |
| Incomplete outcome data | + | + | + | + | + | + | + | + | + |

Risk of bias: +, low; − high~~.~~

Risk of summary: the author’s judgments about each bias item for the included studies.

Risk of bias graph: the author’s judgments about each risk of bias item presented as the percentage across all included studies.

**Figure S3.** Begg’s funnel plot and Egger’s publication bias plots in the meta-analysis of observational studies.

Begg’s Test: *p* for bias = 0.655.

Egger’s Test: *p* for bias = 0.352.

References

1. Moher, D.; Liberati, A.; Tetzlaff, J.; Altman, D.G. Preferred reporting items for systematic reviews and meta-analyses: The PRISMA statement. *PLoS Med.* **2009**, doi:10.1371/journal.
   pmed.1000097.
2. Ackermann, D.; Jones, J.; Comperatore, M.; Volek, J.S.; McIntosh, M.; Kalynych, C.; Vukich, C.; Lott, M.; Fernandez, M.L. Correlation between dietary intake and parameters of the metabolic syndrome in women. *FASEB J.* **2009**, *23*, Available online: http://www.fasebj.org/cgi/content/
   meeting_abstract/23/1_MeetingAbstracts/550.8 (accessed on 15 June, 2014).
3. Gonzalez, E.P.; Santos, F.; Coto, E. Magnesium homeostasis. Etiopathogeny, clinical diagnosis and treatment of hypomagnesaemia. A case study. *Nefrologia* **2009**, *29*, 518–524.
4. Pirpir, A.; Pamukcu, M.; Aksahin, A.; Yildirim, I.S. Association between serum leptin, insulin resistance and blood copper, zinc and magnesium levels in patients with metabolic syndrome. *Obes. Rev.* **2012**, *13*, 650.
5. Villalobos, T.K.; Mascaraque, M.; Andres, P.; Lopez-Sobaler, A.M. Relationship between magnesium intake, c-reactive protein levels and the metabolic syndrome components in a healthy adult population from madrid. *Ann. Nutr. Metab.* **2013**, *63*, 241.
6. Aguilar, M.V.; Saavedra, P.; Arrieta, F.J.; Mateos, C.J.; Gonzalez, M.J.; Meseguer, I.;
   Martinez-Para, M.C. Plasma mineral content in type-2 diabetic patients and their association with the metabolic syndrome. *Ann. Nutr. Metab.* **2007**, *51*, 402–406.
7. Bae, Y.J.; Choi, M.K. Magnesium intake and its relevance with antioxidant capacity in Korean adults. *Biol. Trace Elem. Res.* **2011**, *143*, 213–225.
8. Beydoun, M.A.; Gary, T.L.; Caballero, B.H.; Lawrence, R.S.; Cheskin, L.J.; Wang, Y. Ethnic differences in dairy and related nutrient consumption among us adults and their association with obesity, central obesity, and the metabolic syndrome. *Am. J. Clin. Nutr.* **2008**, *87*, 1914–1925.
9. Bian, S.; Gao, Y.; Zhang, M.; Wang, X.; Liu, W.; Zhang, D.; Huang, G. Dietary nutrient intake and metabolic syndrome risk in chinese adults: A case-control study. *Nutr. J.* **2013**, *12*, 106.
10. Corica, F.; Corsonello, A.; Ientile, R.; Cucinotta, D.; Di Benedetto, A.; Perticone, F.;
    Dominguez, L.J.; Barbagallo, M. Serum ionized magnesium levels in relation to metabolic syndrome in type 2 diabetic patients. *J. Am. Coll. Nutr.* **2006**, *25*, 210–215.
11. De Oliveira, P.B. Study of the magnesium ion in the metabolic syndrome caused by renal insufficiency. I. *Rev. Bras. Med.* **1965**, *22*, 174–180.
12. Folchetti, L.D.; Monfort-Pires, M.; Cezaretto, A.; Barros, C.R.; Ferreira, S.R.G. May zinc and magnesium intakes be useful to indicate oxidative stress, inflammation and insulin resistance in individuals at cardiometabolic risk? *J. Diabetes* **2013**, *5*, 40.
13. Guerrero-Romero, F.; Bermudez-Pena, C.; Rodriguez-Moran, M. Severe hypomagnesemia and low-grade inflammation in metabolic syndrome. *Magnes. Res.* **2011**, *24*, 45–53.
14. Katcher, H.I.; Legro, R.S.; Kunselman, A.R.; Gillies, P.J.; Demers, L.M.; Bagshaw, D.M.;
    Kris-Etherton, P.M. The effects of a whole grain-enriched hypocaloric diet on cardiovascular disease risk factors in men and women with metabolic syndrome. *Am. J. Clin. Nutr.* **2008**, *87*,
    79–90.
15. Kauffman, R.P.; Tullar, P.E.; Nipp, R.D.; Castracane, V.D. Serum magnesium concentrations and metabolic variables in polycystic ovary syndrome. *Acta Obstet. Gynecol. Scand.* **2011**, *90*,
    452–458.
16. Lau Gouveia, E.; Belo, S.; Esteves, C.; Neves, A.C.; Poinhos, R.; Freitas, P.; Varela, A.; Queiros, J.; Correia, F.; Carvalho, D. Hypomagnesemia as a component of metabolic dysfunction in obese. *Obes. Facts* **2012**, *5*, 117.
17. Morrell, J.S.; Lofgren, I.E.; Burke, J.D.; Reilly, R.A. Metabolic syndrome, obesity, and related risk factors among college men and women. *J. Am. Coll. Health* **2012**, *60*, 82–89.
18. Munekage, E.; Takezaki, Y.; Hanazaki, K. Shortage and metabolic disturbance of magnesium in diabetic patients and significance of magnesium replacement therapy. *Clin. Calcium* **2012**, *22*, 1235–1242.
19. Srinivasan, A.R.; Niranjan, G.; Kuzhandai Velu, V.; Parmar, P.; Anish, A. Status of serum magnesium in type 2 diabetes mellitus with particular reference to serum triacylglycerol levels. *Diabetes Metab.* *Syndr. Clin. Res. Rev.* **2012**, *6*, 187–189.
20. Wang, J.; Persuitte, G.; Olendzki, B.C.; Wedick, N.M.; Zhang, Z.; Merriam, P.A.; Fang, H.; Carmody, J.; Olendzki, G.F.; Ma, Y. Dietary magnesium intake improves insulin resistance among non-diabetic individuals with metabolic syndrome participating in a dietary trial. *Nutrients* **2013**, *5*, 3910–3919.
21. Xu, J.; Xu, W.; Yao, H.; Sun, W.; Zhou, Q.; Cai, L. Associations of serum and urinary magnesium with the pre-diabetes, diabetes and diabetic complications in the chinese northeast population.
    *PLoS One* **2013**, doi:10.1371/journal.pone.0056750.
22. Yu, Y.; Cai, Z.; Zheng, J.; Chen, J.; Zhang, X.; Huang, X.F.; Li, D. Serum levels of polyunsaturated fatty acids are low in chinese men with metabolic syndrome, whereas serum levels of saturated fatty acids, zinc, and magnesium are high. *Nutr. Res.* **2012**, *32*, 71–77.
23. Simmons, D.; Joshi, S.; Shaw, J. Hypomagnesaemia is associated with diabetes: Not pre-diabetes, obesity or the metabolic syndrome. *Diabetes Res. Clin. Pract.* **2010**, *87*, 261–266.
24. Lima, M.D.L.; Cruz, T.; Rodrigues, L.E.; Bomfim, O.; Melo, J.; Correia, R.; Porto, M.; Cedro, A.; Vicente, E. Serum and intracellular magnesium deficiency in patients with metabolic
    syndrome-evidences for its relation to insulin resistance. *Diabetes Res. Clin. Pract.* **2009**, *83*,
    257–262.
25. Karar, A.H.; Al-Huwaidi, E.; Lamlums, S.M. Serum phosphorus and magnesium levels in patients with metabolic syndrome. *J. Bahrain Med. Soc.* **2007**, *19*, 56–62.
26. Guerrero-Romero, F.; Rodriguez-Moran, M. Hypomagnesemia, oxidative stress, inflammation, and metabolic syndrome. *Diabetes Metab. Res. Rev.* **2006**, *22*, 471–476.
27. Guerrero-Romero, F.; Rodriguez-Moran, M. Low serum magnesium levels and metabolic syndrome. *Acta Diabetol.* **2002**, *39*, 209–213.
28. Ghasemi, A.; Zahediasl, S.; Syedmoradi, L.; Azizi, F. Low serum magnesium levels in elderly subjects with metabolic syndrome. *Biol. Trace Elem. Res.* **2010**, *136*, 18–25.
29. Evangelopoulos, A.A.; Vallianou, N.G.; Panagiotakos, D.B.; Georgiou, A.; Zacharias, G.A.;
    Alevra, A.N.; Zalokosta, G.J.; Vogiatzakis, E.D.; Avgerinos, P.C. An inverse relationship between cumulating components of the metabolic syndrome and serum magnesium levels.
    *Nutr. Res.* **2008**, *28*, 659–663.
30. Sociedade Brasileira de Cardiologia-SBC; Sociedade Brasileira de Hipertensão-SBH; Sociedade Brasileira de Nefrologia-SBN. V brazilian guidelines of arterial hypertension. *Arq. Bras. Cardiol.* **2007**, *89*, e24–e79 (in Portuguese).
31. Andersen, C.J.; Fernandez, M.L. Dietary strategies to reduce metabolic syndrome. *Rev. Endocr. Metab. Disord.* **2013**, *14*, 241–254.
32. Barbagallo, M.; Dominguez, L.J. Magnesium metabolism in type 2 diabetes mellitus, metabolic syndrome and insulin resistance. *Arch. Biochem. Biophys.* **2007**, *458*, 40–47.
33. Barbagallo, M.; Dominguez, L.J. Magnesium and the cardiometabolic syndrome. *Curr. Nutr. Rep.* **2012**, *1*, 100–108.
34. Barbagallo, M.; Dominguez, L.J.; Galioto, A.; Ferlisi, A.; Cani, C.; Malfa, L.; Pineo, A.; Busardo, A.; Paolisso, G. Role of magnesium in insulin action, diabetes and cardio-metabolic syndrome X.
    *Mol. Asp. Med.* **2003**, *24*, 39–52.
35. Belin, R.J.; He, K. Magnesium physiology and pathogenic mechanisms that contribute to the development of the metabolic syndrome. *Magnes. Res.* **2007**, *20*, 107–129.
36. Champagne, C.M. Magnesium in hypertension, cardiovascular disease, metabolic syndrome, and other conditions: A review. *Nutr. Clin. Pract.* **2008**, *23*, 142–151.
37. Gunther, T. Biochemical mechanisms of the metabolic syndrome and the role of magnesium. *Magnes. Res.* **2010**, *23*, 142–145.
38. Kikuchi, K.; Tanaka, H.; Gima, M.; Kashiwagi, Y.; Shida, H.; Kawamura, Y.; Hasebe, N. Abnormalities of magnesium (Mg) metabolism and therapeutic significance of mg administration in patients with metabolic syndrome, type 2 diabetes, heart failure and chronic hemodialysis.
    *Clin. Calcium* **2012**, *22*, 1217–1226.
39. Lima, M.D.L.; Bomfim, O. Magnesium deficiency on insulin resistance, metabolic syndrome and diabetes mellitus. *Salud Ciencia* **2008**, *15*, 1213–1216.
40. Mooren, F.C.; Krüger, K.; Völker, K.; Golf, S.W.; Wadepuhl, M.; Kraus, A. Oral magnesium supplementation reduces insulin resistance in non-diabetic subjects—A double-blind,
    placebo-controlled, randomized trial. *Diabetes Obes. Metab.* **2011**, *13*, 281–284.
41. Rayssiguier, Y.; Gueux, E.; Nowacki, W.; Rock, E.; Mazur, A. High fructose consumption combined with low dietary magnesium intake may increase the incidence of the metabolic syndrome by inducing inflammation. *Magnes. Res.* **2006**, *19*, 237–243.
42. Rayssiguier, Y.; Libako, P.; Nowacki, W.; Rock, E. Magnesium deficiency and metabolic syndrome: Stress and inflammation may reflect calcium activation. *Magnes. Res.* **2010**, *23*, 73–80.
43. Volpe, S.L. Magnesium, the metabolic syndrome, insulin resistance, and type 2 diabetes mellitus. *Crit. Rev. Food Sci. Nutr.* **2008**, *48*, 293–300.
44. Feldeisen, S.E.; Tucker, K.L. Nutritional strategies in the prevention and treatment of metabolic syndrome. *Appl. Physiol. Nutr. Metab.* **2007**, *32*, 46–60.
45. He, K.; Liu, K.; Daviglus, M.L.; Morris, S.J.; Loria, C.M.; Van Horn, L.; Jacobs, D.R., Jr.;
    Savage, P.J. Magnesium intake and incidence of metabolic syndrome among young adults. *Circulation* **2006**, *113*, 1675–1682.
46. Huang, J.H.; Lu, Y.F.; Cheng, F.C.; Lee, J.N.; Tsai, L.C. Correlation of magnesium intake with metabolic parameters, depression and physical activity in elderly type 2 diabetes patients:
    A cross-sectional study. *Nutr. J.* **2012**, *11*, 41.
47. Al-Daghri, N.M.; Khan, N.; Alkharfy, K.M.; Al-Attas, O.S.; Alokail, M.S.; Alfawaz, H.A.; Alothman, A.; Vanhoutte, P.M. Selected dietary nutrients and the prevalence of metabolic syndrome in adult males and females in saudi arabia: A pilot study. *Nutrients* **2013**, *5*, 4587-4604.
48. Mirmiran, P.; Shab-Bidar, S.; Hosseini-Esfahani, F.; Asghari, G.; Hosseinpour-Niazi, S.; Azizi, F. Magnesium intake and prevalence of metabolic syndrome in adults: Tehran lipid and glucose study. *Public Health Nutr.* **2012**, *15*, 693–701.
49. Park, S.H.; Kim, S.K.; Bae, Y.J. Relationship between serum calcium and magnesium concentrations and metabolic syndrome diagnostic components in middle-aged Korean men.
    *Biol. Trace Elem. Res.* **2012**, *146*, 35–41.
50. Rasic-Milutinovic, Z.; Perunicic-Pekovic, G.; Jovanovic, D.; Gluvic, Z.; Cankovic-Kadijevic, M. Association of blood pressure and metabolic syndrome components with magnesium levels in drinking water in some Serbian municipalities. *J. Water Health* **2012**, *10*, 161–169.
51. Song, Y.; Ridker, P.M.; Manson, J.E.; Cook, N.R.; Buring, J.E.; Liu, S. Magnesium intake,
    C-reactive protein, and the prevalence of metabolic syndrome in middle-aged and older U.S. women. *Diabetes Care* **2005**, *28*, 1438–1444.
52. Bo, S.; Durazzo, M.; Guidi, S.; Carello, M.; Sacerdote, C.; Silli, B.; Rosato, R.; Cassader, M.; Gentile, L.; Pagano, G. Dietary magnesium and fiber intakes and inflammatory and metabolic indicators in middle-aged subjects from a population-based cohort. *Am. J. Clin. Nutr.* **2006**, *84*, 1062–1069.
53. Ford, E.S.; Li, C.; McGuire, L.C.; Mokdad, A.H.; Liu, S. Intake of dietary magnesium and the prevalence of the metabolic syndrome among U.S. Adults. *Obesity* **2007**, *15*, 1139–1146.
54. McKeown, N.M.; Jacques, P.F.; Zhang, X.L.; Juan, W.; Sahyoun, N.R. Dietary magnesium intake is related to metabolic syndrome in older Americans. *Eur. J. Nutr.* **2008**, *47*, 210–216.
55. Noori, N.; Nafar, M.; Poorrezagholi, F.; Ahmadpoor, P.; Samadian, F.; Firouzan, A.; Einollahi, B. Dietary intakes of fiber and magnesium and incidence of metabolic syndrome in first year after renal transplantation. *J. Ren. Nutr.* **2010**, *20*, 101–111.
56. Choi, M.K.; Bae, Y.J. Relationship between dietary magnesium, manganese, and copper and metabolic syndrome risk in korean adults: The Korea National Health And Nutrition Examination Survey (2007–2008). *Biol. Trace Elem. Res.* **2013**, *156*, 56–66.

© 2014 by the authors; licensee MDPI, Basel, Switzerland. This article is an open access article distributed under the terms and conditions of the Creative Commons Attribution license (http://creativecommons.org/licenses/by/4.0/).
